# Potential Use of CRISPR/Cas13 Machinery in Understanding Virus–Host Interaction

**DOI:** 10.3389/fmicb.2021.743580

**Published:** 2021-11-26

**Authors:** Mahmoud Bayoumi, Muhammad Munir

**Affiliations:** ^1^Division of Biomedical and Life Sciences, Lancaster University, Lancaster, United Kingdom; ^2^Virology Department, Faculty of Veterinary Medicine, Cairo University, Giza, Egypt

**Keywords:** CRISPR-Cas, Cas13, RNA interference, RNA-labeling, virus interference, virus resistance, virus diagnosis

## Abstract

Prokaryotes have evolutionarily acquired an immune system to fend off invading mobile genetic elements, including viral phages and plasmids. Through recognizing specific sequences of the invading nucleic acid, prokaryotes mediate a subsequent degradation process collectively referred to as the Clustered Regularly Interspaced Short Palindromic Repeats (CRISPR)–CRISPR-associated (Cas) (CRISPR–Cas) system. The CRISPR–Cas systems are divided into two main classes depending on the structure of the effector Cas proteins. Class I systems have effector modules consisting of multiple proteins, while class II systems have a single multidomain effector. Additionally, the CRISPR–Cas systems can also be categorized into types depending on the spacer acquisition components and their evolutionary features, namely, types I–VI. Among CRISPR/Cas systems, Cas9 is one of the most common multidomain nucleases that identify, degrade, and modulate DNA. Importantly, variants of Cas proteins have recently been found to target RNA, especially the single-effector Cas13 nucleases. The Cas13 has revolutionized our ability to study and perturb RNAs in endogenous microenvironments. The Cas13 effectors offer an excellent candidate for developing novel research tools in virological and biotechnological fields. Herein, in this review, we aim to provide a comprehensive summary of the recent advances of Cas13s for targeting viral RNA for either RNA-mediated degradation or CRISPR–Cas13-based diagnostics. Additionally, we aim to provide an overview of the proposed applications that could revolutionize our understanding of viral–host interactions using Cas13-mediated approaches.

## Introduction

Tugs-of-war usually occur between prokaryotes, including bacteria and archaea against viral phages. As a result, prokaryotes have developed a molecular adaptive immune system to defend against invading viruses. This system is called the Clustered Regularly Interspaced Short Palindromic Repeats (CRISPR)–CRISPR-associated (Cas) (CRISPR–Cas) systems (Makarova et al., 2006). The molecular adaptive system is mainly composed of an effector nuclease(s) encoded by Cas genes and the CRISPR array system. A CRISPR array is usually formed of spacers, which constitute small sequences of invading pathogens to support future degradation. Between these spacers, there are repetitive sequences that act like regulatory elements dubbed as repeats or direct repeats. The spacers and repeats are called CRISPR arrays (Jansen et al., 2002; Yosef et al., 2012; Vercoe et al., 2013).

The process of developing adaptive immunity in the prokaryotes occurs primarily in three steps. The first adaptation step involves the insertion of pathogen-derived sequences in the form of CRISPR arrays (spacers acquisition) (Boyaval et al., 2007). The second maturation step, at which the CRISPR array is transcribed, generates a precursor CRISPR-RNA called (pre-crRNA), which is further processed to create a developed crRNA composed of spacer and repeat sequences (Deltcheva et al., 2011; Jinek et al., 2012). The third interference step involves the addition of mature crRNA to the designated effector nuclease(s) to scan for a complementary seed sequence (Semenova et al., 2011). Upon finding the target sequences, the Cas protein(s) exert a nuclease activity to degrade the target nucleic acid (Wiedenheft et al., 2011). Detailed mechanisms of action for the CRISPR–Cas systems were described elsewhere (Van Der Oost et al., 2014; Marraffini, 2015).

The CRISPR–Cas systems have been identified in some bacterial lineages and archaea (Van Der Oost et al., 2014; Burstein et al., 2016; Burmistrz et al., 2020). These CRISPR–Cas systems vary in crRNA organization and/or the number of the effector Cas proteins. CRISPR–Cas systems can be classified into two classes: Class I usually includes effectors composed of multiple protein subunits, whereas Class II typically carries one single multifunctional effector Cas protein. However, depending on the Cas1–Cas2 evolutionary features, the CRISPR–Cas systems can also be categorized into types, namely, types I–VI. Class I includes types I, III, and IV, whereas Class II includes types II, V, and VI as previously identified (Makarova et al., 2018, 2020). Recently, outstanding achievements have been performed based on the CRISPR–Cas system. It gains this reputation from its higher specificity to target nucleic acids, limited off-target effects, and flexibility to target multiple locations. These criteria are usually needed in biotechnological and molecular biological techniques, including diagnostics and interferences (Brezgin et al., 2019; Banan, 2020). Additionally, the potential use of the CRISPR–Cas system for future vaccine development can be accomplished, as our research group has described earlier (Atasoy et al., 2019; Vilela et al., 2020).

**FIGURE 1:**
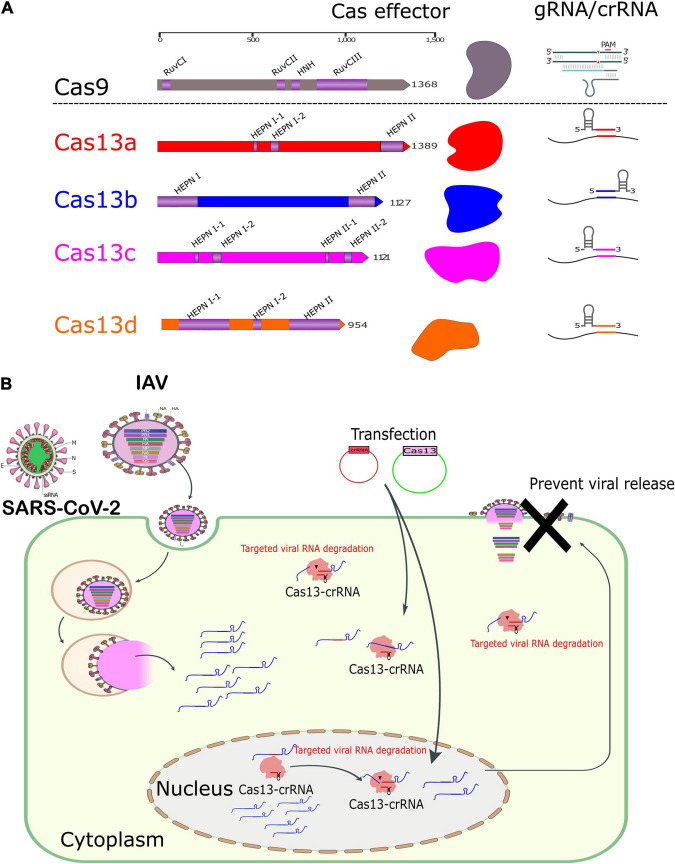
Domain architectures of representative CRISPR effector proteins and RNA-mediated degradation of Cas13s. **(A)** Schematic representation showing the architecture of some proteins of class II CRISPR nucleases. The length of each protein is shown at the top scale. Schematic diagrams for gRNA and crRNAs of the Cas9 and Cas13, respectively, are indicated. **(B)** Schematic representation of the mechanism of action of RNA-mediated degradation of Cas13 nucleases in a cell model.

The majority of research in the past decade has focused on the prototype CRISPR–Cas9 system and its application in the biological fields that usually target DNA that could regulate the genome and possibly epigenome (Soppe and Lebbink, 2017; Banan, 2020). However, the variant endonuclease Cas13 is now well known to target RNA, which could be harnessed for biotechnology and molecular biology (Figure 1A). The concept of the CRISPR–Cas system has been validated for interfering against bacterial viruses (Makarova et al., 2006). The issue made the various research groups validate its use to disturb cellular transcripts, modulate and edit the transcriptome (Abudayyeh et al., 2016; East-Seletsky et al., 2016). Herein, we aim to provide a comprehensive review of the most recent contributions of CRISPR–Cas13 in various biological fields, emphasizing the role of *bona fide* types of CRISPR–Cas13 effectors in virus-related research, especially viral interference and diagnosis. This information can open new avenues for a better understanding of virus–host interactions for future pathogen control and development of novel tools for basic research and biotechnology.

## CRISPR–Cas13 as a Novel Viral Transcriptome-Degradation Method

RNA viruses pose significant threats to all forms of life, including plants, animals, and humans worldwide (Nomaguchi and Adachi, 2017; Woolhouse and Brierley, 2018). Most importantly, human RNA viruses possess potential pandemics, including Zika, Ebola, influenza viruses (Woolhouse and Brierley, 2018), and the contemporary SARS-CoV-2 (causative agent of the COVID-19 disease) (Wu et al., 2020; Zhou et al., 2020). It has been estimated that more than 200 diseases in humans are caused by viruses with RNA genomes (Woolhouse et al., 2012; Woolhouse and Brierley, 2018). These viral diseases contribute to at least 6% of human deaths, as estimated in 2010 (Lozano et al., 2012). However, of these viral diseases and casualties, only nine have approved antivirals, and 15 have licensed vaccines (De Clercq and Li, 2016).

Furthermore, genetic shift, drift, antibody-dependent enhancement, and other immune-mediated diseases complicate the cases of designing ideal vaccines and broad antiviral chemotherapeutics that could take decades to develop, which all provide an antiviral resistance state (Irwin et al., 2016). Despite developing specific monoclonal neutralizing antibodies and small antiviral molecules that are still promising for antiviral therapeutics, high doses of these biological preparations are needed to generate recognizable outcomes. Moreover, increased costs for antiviral production have also implicated this notion (Bai et al., 2012; Kamath, 2016). These problems highlight an urgent need to develop new techniques for combating viral diseases economically and with high sensitivity and specificity and broad activity against multiple viral species. The Cas13 nucleases are novel effectors that could target viral RNA(s) using rationally designed crRNA(s). The successful applications of using CRISPR–Cas13-mediated RNA interference in the various eukaryotic system will be discussed as follows.

### CRISPR–Cas13-Mediated RNA Interference in Plant Viruses

Numerous diseases affect the plant kingdom worldwide, mainly caused by various viruses, and these viruses badly affect crop quality and quantity, which may cause about 50% loss (Fargette et al., 2006; Nicaise, 2014). Specifically for plants, several technologies of pathogen-derived resistance against current plant diseases are available (Baulcombe, 1996; Simón-Mateo and García, 2011; Younis et al., 2014). Furthermore, antiviral gene introduction into crops has a promising capability for engineering resistant crop strains, including R genes (Soosaar et al., 2005). However, these encouraging techniques are confronted with many drawbacks that could limit the usage in plants as reviewed elsewhere (Voinnet, 2005). Therefore, developing new technologies that could interfere with plant viruses is crucial in the near future.

The CRISPR–Cas9 system has been exploited to confer protection against many plant pathogens (Ali et al., 2015, 2016). Additionally, the considerable losses in crops caused by RNA viruses inspired several groups to investigate deployment of CRISPR–Cas13 to interfere with plant viruses (Aman et al., 2018a,b; Mahas et al., 2019; Zhan et al., 2019; Zhang et al., 2019). Upon proofing the concept of using Cas13 effector for targeted RNA degradation (Abudayyeh et al., 2017), Mahfouz’s group was the first to comprehensively harness the prototype CRISPR–Cas13a from *Leptotrichia shahii* (LshCas13a) to interfere with the GFP-labeled turnip mosaic virus (TuMV-GFP) *in planta* (Aman et al., 2018a). The results revealed an almost 50% reduction of the replicating TuMV in the *Nicotiana benthamiana* model (Aman et al., 2018a). Various tested crRNAs directed against multiple locations in the genome of the reporter-expressing virus were assayed. Notably, varying efficiencies in viral CRISPR–Cas13-mediated interference were noticed according to the tested crRNAs (Aman et al., 2018a). The reason behind this variation in viral interference could be the alteration in the secondary RNA structure of crRNA in either transient or stable expression (Abudayyeh et al., 2016; Aman et al., 2018a). Additionally, the processing of Cas13a pre-crRNA arrays into functional units has been validated in plants, which could be harnessed for future multiplexing against various invading viruses (Aman et al., 2018a).

In a follow-up investigation, the same group extends their investigations to corroborate TuMV-GFP viral interference in another plant model using the CRISPR–Cas13 machinery of LshCas13a, *Arabidopsis thaliana*. Viral interference in stable expression in multiple generations was also verified (Aman et al., 2018b). Most importantly, the interference in *A. thaliana* revealed the same level of reduction in GFP and quantified RNA, suggesting that LshCas13a might have reduced efficiency *in planta* (Aman et al., 2018b). These findings lead to a comprehensive screening study to validate and compare various CRISPR–Cas13 orthologs, including Cas13a, Cas13b, and Cas13d, with different localization motifs inside *N. benthamiana* (Mahas et al., 2019). *Leptotrichia wadei* (LwaCas13a) was reported to achieve more efficient activity than the prototype LshCas13a. Additionally, the authors demonstrated a higher efficiency in viral interference of *Prevotella* sp. *P5-125* (PspCas13b) than that of *Bergeyella zoohelcum* (BzCas13b); interestingly, the Cas13d from *Ruminococcus flavefaciens* XPD3002 (CasRx) achieved the highest record of efficiency of viral interference in plants (Mahas et al., 2019). The tested CRISPR machinery showed an absence of collateral activity in the plant (Mahas et al., 2019). Collectively, they established a novel approach with high specificity and deployment to target plant viruses, which could be formulated to target multiple viruses inside the plant cell using various CRISPR–Cas13 effectors (Mahas et al., 2019).

Having demonstrated CRISPR–Cas13a in the plant field, additional research has harnessed LshCas13a to fend off RNA infection in the potato model. With various correctly designed crRNAs, *Potato virus Y* (PVY) was targeted in stable transgenic cell lines that were stably expressing LshCas13a (Zhan et al., 2019). The specificity was performed in closely related viruses, *PVS*, or *PVA* as controls (Zhan et al., 2019). The CRISPR–Cas13a machinery was verified in monocot plants as well, including rice. A piece of evidence shows that LshCas13a can target the dsRNA genome of the *Southern rice black-streaked dwarf virus* (SRBSDV), as investigated previously (Zhang et al., 2019). Collectively, Cas13 orthologs can be utilized efficiently in plants to resist a wide variety of invading pathogens, which could provide the best solutions to improve crop quality along with plant breeding or targeted trait engineering, mainly in developing countries.

### CRISPR–Cas13-Mediated RNA Interference in Animal and Human Viruses

Similar to RNA targeting and degradation *in planta*, the Cas13 nucleases were utilized to perturb RNA in the mammalian cell model (Figure 1B). Bawage et al. (2018) have proposed the use of Cas13a as prevention and therapy to halt both vRNA and mRNA of influenza A virus (IAV) and human respiratory syncytial virus model (RSV). Synthetic mRNA was selected to express *Leptotrichia buccalis* Cas13a (LbuCas13a) instead of using transfecting dsDNA plasmids to avoid genome integration concerns and provide a safe, rapid, and transient action, which will be discussed later in detail. Upon testing of various crRNAs targeting various influenza segments, crRNA targeting the PB1 segment was superior for vRNA and mRNA interference even after 72 h of viral infection, highlighting the prophylactic potential over time (Bawage et al., 2018). The effect of the cleavage Cas13a has no noticeable off-target outcome. Similar findings were validated in previous literature (Abudayyeh et al., 2017; Cox et al., 2017; Bawage et al., 2018; Konermann et al., 2018). Various viral concentrations, timing of viral infection, and cell type showed an equivalent RNA cleavage level, suggesting the treatment possibility upon Cas13 expression. A similar approach was latter applied on the RSV, giving equivalent results (Figure 1B; Bawage et al., 2018).

It has been suggested that Cas13 carries antiviral activity and can be programmed to target various mammalian viruses (Freije et al., 2019). Stepwise investigations were performed to verify the usage of Cas13 orthologs for viral interference. The crRNAs were selected through computational analysis of 396 viral RNAs that correlate with any potential diseases’ progress in humans in a way similar to that adopted for RNA interference (RNAi)-based approaches (von Eije et al., 2008). Two Cas13 orthologs were investigated: *Lwa*Cas13a and *Psp*Cas13b. Three widely separated virus models have been assayed; lymphocytic choriomeningitis virus (LCMV), vesicular stomatitis virus (VSV), and IAV. LCMV was downregulated 2–14-fold using *Lwa*Cas13a. The same conclusions were obtained in IAV and VSV models utilizing *Psp*Cas13b even with different MOIs. Additionally, the comparison between Cas13b and shRNA revealed comparable viral interference, suggesting the possibility of successful future use in viral interference (Freije et al., 2019). However, Cas13 can provide superiority in delivering multiple crRNAs using the CRISPR array. The *Psp*Cas13b activity has been evaluated using four different crRNAs successfully, highlighting the Cas13 technology for targeting multiple viruses *in vivo* (Freije et al., 2019). Furthermore, localization of Cas13 expression was reported to be an essential factor for optimal antiviral infectivity. The Cas13b version expressed in the cytosol has significant antiviral activity compared with the nuclear-expressed version, highlighting that the mRNA of the influenza virus was intensively targeted upon the cytoplasmic expression of Cas13b (Freije et al., 2019). Moreover, the supernatant viruses’ sequencing reveals the absence of mutations at the crRNA target sequence after Cas13-mediated activity, highlighting the lower possibility of viral adaptive mutations upon Cas13 expression and activity (Freije et al., 2019).

Recently, upon the prevalence of COVID-19, many scientists raced to develop antiviral approaches to combat the global pandemic. Abbott et al. (2020) have developed a CRISPR–Cas13d-based system as a prophylactic method against SARS-CoV-2 infection, dubbed as the prophylactic antiviral CRISPR in human cells (PAC-MAN) strategy using Cas13d. The Cas13d is usually retrieved from the *R. flavefaciens* XPD3002 and utilizes a 22-nucleotide crRNA spacer for target RNA degradation (Konermann et al., 2018). Notably, among Cas13 nucleases, the Cas13d ortholog exhibited superiority in its targeted degradation among various tested CRISPR–Cas13 machineries identified so far (Konermann et al., 2018; Mahas et al., 2019). The PAC-MAN approach was assayed against the synthetic RNA of SARS-CoV-2- and IAV-infected lung epithelial cells (Abbott et al., 2020). Both transient and stable cells expressing Cas13d and crRNA were confirmed to degrade the synthetic-labeled RNA fragments in the presence of adequately designed crRNA, which was essential to achieve maximum interference (Abbott et al., 2020). Testing of IAVs using a similar approach was performed by targeting the end of viral segment utilizing a high MOI for infection (Abbott et al., 2020); this approach differs from those targeting the conserved sequence inside viral segments using a low MOI (Freije et al., 2019). However, both exerted a significant viral degradation, testifying to the potential of this approach as an antiviral strategy.

Very recently, the Santangelo group complemented their approach described before (Bawage et al., 2018) for utilizing the mRNA expressing Cas13a for prophylaxis and treatment of COVID-19 and flu diseases (Blanchard et al., 2021). Fascinatingly, they verified their Cas13 methodology using an animal model for the first time for treating viral infections so far. Notably, both cytosolic and nuclear-expressed versions showed a reduction in PB1 transcribed RNA that differs from what was found in Cas13b targeting NP, as stated before (Freije et al., 2019). It appeared that the crRNA against the target sequence was the cause of this notion. Notably, careful selection of RNA target sites and exploration of various crRNAs’ efficiency might be essential to maximize the Cas13 effector’s activity and the success of the Cas13-mediated RNA interference.

In comparison to an established siRNA system against the influenza virus, superior interference of Cas13a was noticed. Very recently, it has been identified that an epigenetic force controls siRNA specificity and potency (Rydzik et al., 2021). The activity was maximized in the presence of a combination of crRNA target PB2 transcripts (Blanchard et al., 2021). Additionally, authors evaluated the interference over time and found that after Cas13a expression, the influenza virus replication was downregulated over 3 days post transfection. Similar viral inhibition was detected in testing SARS-CoV-2-infected VeroE6 cells using crRNA targeting the replicase and nucleocapsid transcripts (Blanchard et al., 2021). The success of viral degradation in cell models encourages them to fetch the Cas13 nuclease performance in animal models. The CRISPR–Cas13 machinery was delivered along with promising guides in a nano-based polymer (poly-beta-amino-esters nano-vehicles based, PBAE) (Patel et al., 2019). Notably, influenza virus reduction was verified in the animal model, suggesting a robust knockdown of influenza *in vivo*. A similar finding was achieved in hamsters with infection by SARS-CoV-2 (Blanchard et al., 2021). A similar success of using Cas13a was reported against hepatitis disease virus (HCV), suggesting an excellent antiviral activity against chronic diseases (Ashraf et al., 2021).

*Vis-à-vis* Cas13’s success for an animal viral model, porcine reproductive and respiratory syndrome virus (PRRSV) was verified (Cui et al., 2020). Owing to their maximal RNA knockdown effect, authors decided to use the *Psp*Cas13b ortholog compared with an array of Cas13a, Cas13b, and Cas13c effectors (Cox et al., 2017; Cui et al., 2020). RNA cleavage was assayed primarily using a synthetic plasmid expressing ORF7 (nucleocapsid protein) fused with eGFP. They noticed that a transient expression of ORF7 along with a single vector expressing Cas13b and crRNA leads to about 60% RNA degradation (Cui et al., 2020). Furthermore, the generation of an all-in-one system that can express both Cas13b and two crRNAs for multiplexing was confirmed to attain superior RNA interference (Cui et al., 2020). Additionally, they confirmed this finding after stable expression of the all-in-one system in MARC-145 cell, achieving an almost complete degradation of the PRRSV genome (Cui et al., 2020). All these conclusions support the successful use of Cas13 proteins in controlling viral infection in the future, in either prophylactic or treatment conditions, and importantly, in engineering mammalian cells that stably express Cas13 effectors and poly-crRNAs targeting multiple viruses and could provide stable immunity *in vitro*. This approach could be used as a first step for a future generation of transgenic animals that resist enzootic diseases and for targeted antiviral therapeutic in humans. However, comprehensive investigation of safety-related issues and immunogenicity *in vivo* are of paramount importance prior to any field applications.

## Fundamental Concerns of Using Cas13 Effectors for Targeted Antiviral Therapeutics

Given the aforementioned modalities and proof-of-concept use of Cas13 nucleases in different eukaryotic systems, Cas13s could be poised shortly to cure human viral diseases. Importantly, using Cas13 ribonucleases for targeted antiviral therapeutics *in vivo* would raise pivotal concerns toward potential risks of administering Cas13s in human bodies. How these bacterial-derived macromolecules would engage with the immune system should be answered before any future administration in animals and/or humans. Learning from the lesson of the prototype CRISPR–Cas9s and the variant Cas12, we could expect that Cas13-targeted therapeutics have to pass the main challenges facing any enticing technology from the laboratory to clinical translation. The main challenges facing this notion includes high specificity, accurate delivery, lower immunogenicity, and long-lasting effect to the inoculated *in vivo* model (Chew, 2018). Accordingly, various groups have utilized Cas13s with an exquisite level using rationally designed crRNA(s) to target one or more viruses (Aman et al., 2018a; Cui et al., 2020; Blanchard et al., 2021).

After the specificity testing of the Cas13s is passed, safe and efficient delivery to the designated cells and tissue *in vivo* is crucial for their maximal functionality. Therefore, various viral and non-viral methods need to be utilized for this notion (Yin et al., 2016; Wang et al., 2017). Viral delivery is considered the most common method to administer recombinant DNA either *in vitro* and *in vivo*. However, careful selection should be considered from the possibility of integration and adverse immune reaction. Vector-induced cytotoxicity has been reported against administered Cas9 carried on the adenoviral vector (Wang et al., 2015). Incompetent adeno-associated viral (AAV) vectors were also utilized with lower immunogenicity *in vivo* with reduced cytotoxic outcomes (Yang et al., 2016; Jarrett et al., 2017). In this context, utilization of Cas13d would be leveraged for their superior specificity compared with other Cas13s and small size that could be integrated easily in a viral vector (Konermann et al., 2018; Mahas et al., 2019). Non-viral delivery has also been utilized for various genome-editing nucleases including hydrodynamic injection, cell penetration peptides, various nanoparticles, and electroporation with varying efficiency as reviewed elsewhere (Wang et al., 2017).

However, developing novel and safe technologies for targeted delivery of Cas13s *in vivo* warrants further investigations. In this milieu, purified ribonucleoprotein (RNP) delivery could be promising for delivery that eliminates any potential risk of genome integrations as reported in Cas9s (Kim et al., 2014). The development of synthetic mRNA could provide a magic clue as well to prevent possible DNA integration. This concept was proved; as discussed earlier, the synthetic mRNA of Cas13a was helpful for prophylactic and therapeutic effects against IAV and SARS-CoV-2 in both cells and animal models (Bawage et al., 2018; Blanchard et al., 2021). Notably, the contemporary COVID-19 is a salient successful example to demonstrate the use of mRNA vaccines safely in humans (Borah et al., 2021). Notably, the synthesized mRNA could be modulated to support lower immunogenicity to the inoculated animal/human bodies (Andries et al., 2015; Loomis et al., 2018).

*Vis-à-vis* immunogenicity of Cas13s *in vivo*, growing pieces of evidence support the presence of preexisting immunity against Cas9 and Cas12 in current clinical trials for targeted genome-editing and cancer therapeutics (Chew, 2018; Crudele and Chamberlain, 2018; Gough and Gersbach, 2020; Mehta and Merkel, 2020). Thus, similar anti-Cas13 antibodies and cellular immune response could be predicted as well from clinical translational studies. Importantly, both systems share a similar protein length of their single multifunctioning protein (Figure 1A). However, the desired targeted genome-editing function by Cas9 was successfully executed even in the presence of specific immune responses against Cas9 (Wang et al., 2015; Chew, 2018). Additionally, careful dissection between the adverse effects of some delivery methods and the efficiency of the CRISPR system should be considered.

Considering the common bacterial load around us naturally, it is unswerving that the immune system can mount a response against large proteins like Cas9 and Cas13. Additionally, this immune response did not abrogate the targeted genome-editing function and possibly the antiviral therapeutics. Therefore, proteins with reduced immunogenicity would be helpful to empower the use of Cas13-mediated genome editing (Wignakumar and Fairchild, 2019; Wagner et al., 2021). Despite microbial-derived Cas13s lacking many post-translational modifications to mount a detrimental immune response, adopting humanization strategies of Cas proteins through masking bacterial epitopes with maintaining the functionality of the proteins would be a clue for evading innate immunity (Chew, 2018). Importantly, if the immune system response is inevitable, the evasion of the immune system is a prerequisite. Various methods could be adopted to evade immunity including adding decoy antigens to alleviate the innate immunity, which, in turn, could minimize the adaptive response (Symons et al., 1995; Wignakumar and Fairchild, 2019). Furthermore, computational analysis of immune system reaction against the targeted therapeutic proteins could be predicted through a list of *in silico* tools to avoid strong responses (Chew, 2018).

Notably, Cas13 research is still considered in its infancy compared with Cas9 in terms of clinical testing. Thus, additional research to address clinical use warrants investigations to enrich our understanding of the potential risks of using the CRISPR–Cas system for any clinical translation. Collectively, understanding many clues in the immune response against Cas13 could help us to not only avoid keeping this enticing technology to be on hold, which could terminate the technology early, but also preserve the long-term efficiency of a viable solution against devastating animal and human diseases.

## CRISPR–Cas13 as a Novel Viral Diagnostic Platform

Continuous emerging and re-emerging of pathogens that affect humans and livestock animals possess the threats of causing epidemics and potential pandemics, which eventually affect global security. These myriad of pathogens highlight the need to develop various diagnostic assays for surveillance, epidemiological studies, and biotechnological investigations. Importantly, the current commercial diagnostic assays are usually set off between multiple criteria, including the analytical sensitivity, off-target effect, field-deployment capability, cost-effectiveness, speed, simplicity, multitarget detection, and readout formats. Therefore, developing an ideal platform that could provide the advantages of these criteria mentioned above is a major obstacle confronting scientists. The microbial adaptive immune system CRISPR–Cas13-guided RNases offer an unprecedented advantage in bacteria as a defense mechanism and for diagnosis *in vitro*. Upon degrading the target sequence, the Cas13 promiscuously cuts into the adjacent non-target RNA that is referred to as collateral activity, which is supposed to respond to subsequent programmed cell death (Abudayyeh et al., 2016). The Cas13 programmed collateral activity was leveraged *in vitro* to degrade a synthetic-labeled non-target RNA (reporter).

**FIGURE 2:**
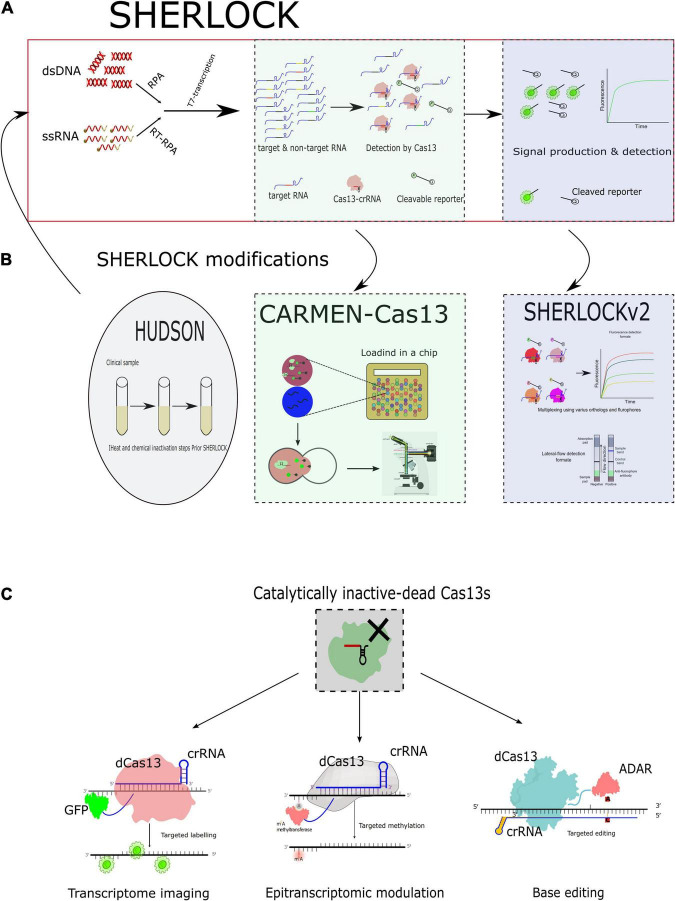
CRISPR–Cas13-based diagnostics and applications of the catalytically inactive Cas13s (dCas13s) in virological applications. **(A)** Schematic diagram for Specific High-Sensitivity Enzymatic Reporter UnLOCKing (SHERLOCK). RPA, recombinase polymerase amplification. **(B)** Schematic diagrams for the modifications added to the SHERLOCK. HUDSON, heating unpurified diagnostic samples to obliterate nucleases; the HUDSON was usually performed before the prototype SHERLOCK. CARMEN, combinatorial arrayed reactions for multiplexed evaluation of nucleic acids; the emulsions were added to chip for detection, and the detection is usually occurs using fluorescence-based microscopy. SHERLOCKv2 differs from the prototype in using various Cas13 effectors represented by different color-coded proteins. SHERLOCKv2 varies in readout format as well as in lateral flow format. **(C)** Schematic representation of the possible applications of the catalytically inactive Cas13 (dCas13s) fusion for imaging, editing, and modulation.

Gootenberg et al. (2017) were the first to harness the CRISPR–Cas13 to be used for diagnosis purposes through developing a platform capable of providing attomolar (aM) sensitivity with enhanced specificity identified as the Specific High-Sensitivity Enzymatic Reporter Unlocking (SHERLOCK). The SHERLOCK is a technique that combines one of the isothermal assay techniques (recombinase polymerase amplification, RPA) with the collateral activity of a single effector protein, Cas13 (Gootenberg et al., 2017). RNA sensing was attained by reprogramming the single-effector RNA-guided ribonucleases (RNases) LwaCas13a to degrade labeled synthetic RNA; it has been reported that LwaCas13a provides a more robust signal than LshCas13a (Abudayyeh et al., 2016). Authors improved sensitivity using RPA to provide prior amplification and T7 transcriptase to generate RNA from the amplified DNA to be liable for targeting by Cas13, which could detect single-molecule input (Figure 2A; Gootenberg et al., 2017). Moreover, the technique tolerates lyophilization and rehydration without affecting reaction components and sensitivity for further field deployment (Gootenberg et al., 2017). Furthermore, the platform could benefit in genotyping human diseases through spanning single-nucleotide polymorphism and identification of various cancers as well (Gootenberg et al., 2017).

In a follow-up study, the same group improved a SHERLOCKv2 platform to include many advantages to clinical diagnostics. Authors have leveraged various Cas13 orthologs that differ in their specific cleavage sequence preferences for in-sample multiplexing (Gootenberg et al., 2018). They successfully confirmed a quadruplex assay in a single tube using different orthologs and detection channels to detect inputs to an exquisite sensitivity level (Gootenberg et al., 2018). Furthermore, the authors leveraged additional unrelated CRISPR type III nuclease called Csm6 to provide enhancement of the detection signal. Csm6s are usually activated by the presence of short oligoadenylates with 2′,3′-cyclic phosphate ends (Jiang et al., 2016; Gootenberg et al., 2018; Liu et al., 2021). Combining Cas13 and Csm6s with hexadenylates containing 2′,3′-cyclic phosphate ends achieved a 3.5-fold increase in signal sensitivity (Gootenberg et al., 2018). The approach of tandem enzyme activation was further improved to achieve direct RNA sensing with accelerated detection through combining Cas13 and Csm6 with a chemically stabilized activator to detect around 30 RNA/μL molecules in 20 min (Liu et al., 2021). Collectively, these achievements could potentiate the portability of Cas13-based approaches at point-of-care testing with higher sensitivity, portability, and multiplexing capabilities.

In case of emerging and re-emerging viral diseases, high sensitivity, specificity, and out-of-lab diagnostics are urgently needed. Moreover, infections that have similar clinical outcomes with closely related viral causes could complicate this notion. Therefore, Myhrvold et al. (2018) have harnessed the high specificity and sensitivity of the Cas13-based diagnostic platform, SHERLOCK, to be primarily used for viral diagnosis directly from patients in out-of-lab settings utilizing the lateral-flow readout. Their modified platform combined SHERLOCK assay and HUDSON (heating unextracted diagnostic samples to obliterate nucleases). Through this approach, the viral particles that were released from bodily fluids (e.g., urine, saliva, whole blood, serum, and plasma) were lysed and inactivated with chemical and thermal processing in instrument-free settings (Myhrvold et al., 2018). The genetically similar viruses, including ZIKV, WNFV, YFV, and DENV, were differentiated with high sensitivity and the lowest off-target effect in less than 2 h in a field-deployable setting (Myhrvold et al., 2018). Additionally, successful detection and identification of various single-nucleotide polymorphisms (SNPs) and drug-resistant variants of HIV and ZIKV were demonstrated to be combined in the HUDSON-SHERLOCK platform (Figure 2B; Myhrvold et al., 2018).

Recently, the Sabeti group combined the use of CRISPR–Cas13 with CARMEN (Combinatorial Arrayed Reactions for Multiplexed Evaluation of Nucleic acids). The platform enabled the detection of multiplexed pathogens (Ackerman et al., 2020). Depending on microfluidics technology, they were able to multiplex viral detection in a miniaturized format. Through this platform, the operator will need to mix preamplified nucleic acids of interest. The amplified nucleic acids will be introduced to the test in an emulsion form along with the emulsified Cas13 detection mix (i.e., crRNA and reporter) in a color-coded context. After all were mixed in one tube, these pooled emulsion droplets are loaded in the microfluidic chip (Ackerman et al., 2020). This chip contains thousands of paired wells. The emulsions are merged with forming all possible combinations with the various crRNAs to give fluorescence readout in case of positive detection, which is detected using fluorescent-based microscopy (Figure 2B; Ackerman et al., 2020). Despite CARMEN–Cas13 maintaining the high sensitivity of viral detection as that found for SHERLOCK- and PCR-based detection, it surpasses others in the multiplexing capability to test at least 1,000 samples using single microfluidic chips (Ackerman et al., 2020).

Successful use of SHERLOCK-containing platforms encourages various groups to deploy them for the diagnosis of life-threatening viral hemorrhagic fevers, including the Ebola virus (EBV) (Qin et al., 2019; Barnes et al., 2020). EBV usually requires higher biosafety levels for handling (Feldmann et al., 2003). Inactivation of infectious material inputs was performed using the HUDSON technique, and then RNA sensing using the SHERLOCK assay was performed in both fluorescent and visual readout formats. Additionally, screening the outbreaks using a mobile application in various infected countries was also applicable (Barnes et al., 2020). SHERLOCK was also applied to detect the contemporary SARS-CoV-2 in a short time (Joung et al., 2020; Abudayyeh and Gootenberg, 2021). Collectively, the platforms mentioned above and their improved versions highlight the successfulness of the guided RNases in viral diagnostic-based assays and biotechnological applications and suggest further improvements to develop an ideal approach for diagnosis (Abudayyeh and Gootenberg, 2021; Shinoda et al., 2021). A summary of the successful applications of Cas13 effectors in viral RNA degradation and diagnosis is listed in Table 1.

**TABLE 1:** Summary of the Cas13 effectors and their applications for viral RNA degradation and CRISPR-based diagnostics.

Application	Viruses	Cas13 orthologs	References
**A. RNA degradation**			
	Turnip mosaic virus (TuMV)	*Lsh*Cas13a	Aman et al., 2018a,b
	Turnip mosaic virus (TuMV) Tobacco mosaic virus (TMV)-RNA-based overexpression (TRBO-G)	*Lwa*Cas13a *Psp*Cas13b CasRx	Mahas et al., 2019
	Potato virus Y (PVY)	*Lsh*Cas13a	Zhan et al., 2019
	Southern rice black-streaked dwarf virus (SRBSDV)	*Lsh*Cas13a	Zhang et al., 2019
	Influenza A virus (IAV) Respiratory syncytial virus model (RSV)	*Lbu*Cas13a	Bawage et al., 2018
	Lymphocytic choriomeningitis (LCMV) Vesicular stomatitis virus (VSV) IAV	*Lwa*Cas13a *Psp*Cas13b	Freije et al., 2019
	Severe acute respiratory syndrome coronavirus 2 (SARS-CoV-2) IAV	CasRx	Abbott et al., 2020
	SARS-CoV-2 IAV	*Lbu*Cas13a	Blanchard et al., 2021
	Hepatitis C virus HCV	*Lsh*Cas13a	Ashraf et al., 2021
	Porcine reproductive and respiratory syndrome virus (PRRSV)	*Psp*Cas13b	Cui et al., 2020

**B. CRISPR-based diagnosis**			
	ZIKA and Dengue RNA (SHERLOCK)	*Lwa*Cas13a	Gootenberg et al., 2017
	ZIKA and Dengue RNA (SHERLOCKv2)	*Lwa*Cas13a, *Cca*Cas13b *Lba*Cas13a *Psm*Cas13b	Gootenberg et al., 2018
	Flaviviruses (HUDSON-SHERLOCK)	*Lwa*Cas13a	Myhrvold et al., 2018
	All 169 human-associated viruses, Including IAV, SARS-CoV-2, HIV (CRISPR-Cas13 with CARMEN)	*Lwa*Cas13a	Ackerman et al., 2020
	Ebola virus (EBV) and Lassa virus (SHERLOCK-HUDSON)	*Lwa*Cas13a	Qin et al., 2019; Barnes et al., 2020

## Future Potential Applications of CRISPR–Cas13 Effectors for Better Understating the Virus–Host Interaction

Given the aforementioned successful usage of the CRISPR–Cas13 system in microbial diagnosis, RNA interference, and various molecular biology applications, new avenues that Cas13 effectors can achieve soon to better understand virus replication kinetics and virus–host interplay are suggested as follows.

### Mapping Intracellular Localization of the Viral Genome

Intracellular protein labeling is usually attained by linking with either fluorescent proteins or dyes. In contrast, intracellular transcript imaging is an ambitious target to study RNA dynamics. Until recently, the fluorescence *in situ* hybridization technique (FISH) was predominantly used to explain the intracellular RNA dynamics in fixed cell models (Raj et al., 2008; Alonas et al., 2016). The breakthrough, which have performed to understand the prototype Cas13 function, highlighted also that Cas13 could label specific transcripts including *ACTB* utilizing the catalytically inactive form of Cas13 (dCas13) (Abudayyeh et al., 2017). Inactivation is usually achieved by inducing mutation in the putative catalytic arginine residues within the HEPN domains. RNA-binding assessment has been compared with the standardized method for targeting transcripts in fixed samples, FISH (Abudayyeh et al., 2017). Regardless of the genome nature of the viruses, all use RNA as an intermediate molecule for various functions, including protein translations. Therefore, unraveling the localization, transportation, nuclear exportation of some viruses, assembly, and dynamics of viral RNA is pivotal in underpinning the fundamental role and interaction of viruses with the cellular machinery (Figure 2C). Additionally, this imaging will likely open new frontiers for generations of antivirals.

Recent RNA invasive labeling techniques validate tracking cellular RNA in many organisms, including the bacteriophage coat protein (MS2)-based reporter system, spinach, broccoli, and Cas9 designed for RNA targeting (RCas9) (Briley et al., 2015; Miorin et al., 2016; Nelles et al., 2016; Yang and Chen, 2017; Rauch and Dickinson, 2018; Yang et al., 2019; Liu et al., 2020). Specifically, in virology, the MS2-based system was previously used for tracking HIV in various transcription and assembly processes (Jouvenet et al., 2009; Sardo et al., 2015). Furthermore, multiply labeled tetravalent RNA imaging probes (mTRIP) were used for visualizing RSV (Alonas et al., 2016). Besides, others used fluorescence recovery after photobleaching (FRAP) and fluorescence loss in photobleaching (FLIP) for labeling various flaviviruses (Miorin et al., 2016). However, due to the limitations of the intracellular RNA labeling techniques that affect cellular and possibly viral RNA dynamics as noticed in the MS2-based system or short-time tracking process, novel labeling techniques are of paramount importance (Alonas et al., 2016; Yang et al., 2019).

A recent systematic investigation comparing the Cas13 orthologs and the most common method of RNA labeling, the MS2-based labeling, was performed. The study revealed that the Cas13s outperform the labeling efficiency of the MS2-based system and that dPspCas13b was the most efficient ortholog without affecting cellular transcript dynamics (Yang et al., 2019; Han et al., 2020). They also validated using various Cas13 orthologs simultaneously, which could open the way to target more than one transcript as segmented RNA viruses, including influenza (Yang et al., 2019; Ahmed et al., 2020). Additionally, combining the catalytically inactive Cas13 (dCas13) with the dCas9 version could open new avenues for tracking RNA and DNA simultaneously in DNA-replicating viruses, including herpesvirus models (Yang et al., 2019; Bayoumi et al., 2020a,^2021^).

### Base Editing of Viral Transcriptome

Similar to the investigations performed for validation using Cas13 effectors for targeted RNA degradation and binding (labeling) (Abudayyeh et al., 2016, 2017). The same group performed further investigations reporting possible usage of Cas13 effectors for targeted editing to the transcriptome as well (Cox et al., 2017). They fused dead (inactive) Cas13 effectors with the adenosine deaminase acting on RNA type 2 (ADAR2). This approach yielded a high specificity of binding and efficiency for editing adenosine to inosine in cellular transcripts. The method for editing is called RNA Editing for Programmable A to I Replacement (REPAIR) (Figure 2C; Cox et al., 2017). This promising approach could alleviate the inherent genetic-based mutations by producing fully functional proteins via editing the intermediate RNA level instead of introducing exogenous functional proteins that usually have aberrant outcomes with enhanced immunogenicity (Qu et al., 2019). The system has high binding editing efficiency with unnoticeable off-target effects all over the transcriptome (Cox et al., 2017; Qu et al., 2019). The Cas13 targeted editing system had a broad spectrum of applications, including in various mammalian cells (Cox et al., 2017; Qu et al., 2019) and yeasts (Jing et al., 2018).

### Epigenetic Modification of Viral Replication and Protein Expression

Moreover, the Cas13-based RNA editing approach was recently repurposed to generate epitranscriptome modifications to cellular transcripts via fusing dCas13 effectors with various m6A-related proteins (Li et al., 2020; Wilson et al., 2020; Zhao et al., 2020; Xia et al., 2021). The m6A is an epitranscriptomic mark that controls multiple aspects of viral replication and the outcomes of virus–host interactions. Thus, the manipulation of viruses intracellularly could be achieved through RNA editing as well (Figure 2B; Kennedy et al., 2017; Bayoumi et al., 2020b; Tsai and Cullen, 2020; Bayoumi and Munir, 2021a,b). Given the aforementioned successful application for RNA editing, the Cas13s can be repurposed to induce specific mutations to viral transcripts to improve viral intervention approaches shortly. Similarly, editing of more than one segment utilizing various orthologs can be attained. In this way, we could manipulate viral RNA both genetically and epigenetically. Cas13 effectors were utilized very recently to study circular RNAs driven from cellular transcripts, which could be exploited for use in circRNAs containing viruses shortly (Nahand et al., 2020; Li et al., 2021).

### Modulation of Viral RNA–Protein Interaction, Alternative Splicing, and Polyadenylation

As an obligate intracellular parasite, viruses are dependent on majority of cellular machinery to establish competent replication. Therefore, the identification and characterization of various interactions of viral RNAs with the cellular proteins could revolutionize our understanding of viral RNA function (Girardi et al., 2021). Hence, we can develop potential antiviral ways to block the vRNA interaction with those proteins that have a proviral effect (Schmidt et al., 2021). dCas13 effectors have been verified to block cellular transcript RNA–protein binding sites targeted by crRNA (Yao et al., 2019). In this way, viral RNA binding sites can be blocked to inhibit downstream function. Alternative splicing is another aspect that viruses can benefit from to expand their proteome from the smaller genome. However, maintaining a specific ratio between various protein isoforms is essential for efficient viral replication (Dubois et al., 2014; Artarini et al., 2019). Additionally, Cas13 has been fused efficiently with various splicing factors to drive alteration in exon exclusion and inclusion in the cellular transcriptome (Du et al., 2020; Leclair et al., 2020). Therefore, modulation of viral replication through alteration of alternative splicing could be a tool for studying viral replication kinetics and potential antiviral strategy. Similarly, the viral infection could be modulated by alteration in the polyadenylation state of viral transcripts intracellularly, through binding the Cas13 with polyadenylation factors to drive cleavage and/or polyadenylation accordingly (Pritlove et al., 1998; Anderson et al., 2019). Collectively, Cas13 effectors could enrich our understanding of viral RNA function and the interplay of the virus–host interaction.

### Repurposing the Immune System Against Viral Replication and Protein Expression

Given the aforementioned applications of Cas13 effectors through interaction and inhibition of viral replication directly, controlling the viral infection could be achieved indirectly through repurposing the immune system to engage actively and efficiently, such as rescuing MHC class I/II transcript expression, and modulating the activation or suppression of cytokines accordingly, which could alter specific T lymphocytes to respond to certain antigens rapidly. This approach will greatly improve host innate immunity and subsequently adaptive immunity as previously adopted for enhancing antitumor immune response (Alves et al., 2021). Finally, it should be noted that several recently identified Cas13 proteins have not been investigated yet. Comprehensive investigations of Cas13c effectors are still scarce so far, which could revolutionize our ability to perturb RNA and provide additional clues and support various biotechnological applications, including multiplexing, RNA binding, and RNA-mediated degradation (Shmakov et al., 2017; Palaz et al., 2021). These successful applications collectively prove that CRISPR–Cas systems should be exploited massively for viral diagnosis/degradation, understanding of virus replication in depth, and development of various Cas13-based viral manipulation strategies in the near future.

## Author Contributions

MM: conceptualization and writing—review and editing and supervision. MB and MM: formal analysis and investigation and resources. MB: writing—original draft preparation. Both authors contributed to the article and approved the submitted version.

## Conflict of Interest

The authors declare that the research was conducted in the absence of any commercial or financial relationships that could be construed as a potential conflict of interest.

## Publisher’s Note

All claims expressed in this article are solely those of the authors and do not necessarily represent those of their affiliated organizations, or those of the publisher, the editors and the reviewers. Any product that may be evaluated in this article, or claim that may be made by its manufacturer, is not guaranteed or endorsed by the publisher.
